# Gene-Specific Effects on Brain Volume and Cognition of *TMEM106B* in Frontotemporal Lobar Degeneration

**DOI:** 10.1212/WNL.0000000000209832

**Published:** 2024-09-25

**Authors:** Marijne Vandebergh, Eliana Marisa Ramos, Nick Corriveau-Lecavalier, Vijay K. Ramanan, John Kornak, Carly Mester, Tyler Kolander, Danielle E. Brushaber, Adam M. Staffaroni, Daniel H. Geschwind, Amy A. Wolf, Kejal Kantarci, Tania Gendron, Leonard Petrucelli, Marleen Van den Broeck, Sarah Wynants, Matthew Baker, Sergi Borrego-Écija, Brian Appleby, Sami Barmada, Andrea C. Bozoki, David Clark, R. Ryan Darby, Bradford C. Dickerson, Kimiko Domoto-Reilly, Julie A. Fields, Douglas Galasko, Nupur Ghoshal, Neill R. Graff-Radford, Ian M. Grant, Lawrence S. Honig, Ging-Yuek R. Hsiung, Edward D. Huey, David J. Irwin, David S. Knopman, Justin Y. Kwan, Gabriel C. Léger, Irene Litvan, Joseph C. Masdeu, Mario F. Mendez, Chiadi U. Onyike, Belen Pascual, Peter S. Pressman, Aaron Ritter, Erik D. Roberson, Allison Snyder, Anna Campbell Sullivan, Maria Carmela Tartaglia, Dylan Wint, Hilary W. Heuer, Leah K. Forsberg, Adam L. Boxer, Howard J. Rosen, Bradley F. Boeve, Rosa Rademakers, for the ALLFTD Consortium

**Affiliations:** From the VIB Center for Molecular Neurology (M.V., R.R., V.B., S.W.); Department of Biomedical Sciences (M.V., M.V.B., S.W., R.R.), University of Antwerp, Belgium; Department of Neurology (E.M.R., M.F.M.), David Geffen School of Medicine, University of California, Los Angeles; Department of Neurology (N.C.-L., V.K.R., T.K., K.K., B.F.B.); Department of Psychiatry and Psychology (N.C.-L., J.A.F., D.S.K., L.K.F.), Mayo Clinic, Rochester, MN; Department of Epidemiology and Biostatistics (J.K.), University of California, San Francisco; Department of Quantitative Health Sciences (C.M., D.E.B.), Mayo Clinic, Rochester, MN; Department of Neurology (A.M.S., A.A.W.), Memory and Aging Center, University of California, San Francisco; Weill Institute for Neurosciences, San Francisco, California; Institute for Precision Health (D.H.G.), Departments of Neurology, Psychiatry and Human Genetics at David Geffen School of Medicine, UCLA; Department of Neuroscience (T.G., L.P., M.B., N.R.G.-R.), Mayo Clinic, Jacksonville, FL; Alzheimer's Disease and Other Cognitive Disorders Unit (S.B.-É.), Neurology Service, Hospital Clínic de Barcelona, Institut d'Investigacions Biomèdiques August Pi i Sunyer (IDIBAPS), Fundació Clínic per a la Recerca Biomèdica, Uni; Department of Neurology (B.A., B.C.D.), Case Western Reserve University, Cleveland, OH; Department of Neurology (S.B.), University of Michigan, Ann Arbor; Department of Neurology (A.C.B.), University of North Carolina, Chapel Hill; Department of Neurology (D.C.), Indiana University, Indianapolis; Department of Neurology (R.R.D.), Vanderbilt University, Nashville, TN; Department of Neurology (K.D.-R.), University of Washington, Seattle, WA; Department of Neurosciences (D.G., G.C.L., I.L.), University of California, San Diego, La Jolla; Departments of Neurology and Psychiatry (N.G.), Washington University School of Medicine, Washington University, St. Louis, MO; Department of Psychiatry and Behavioral Sciences (I.M.G.), Northwestern Feinberg School of Medicine, Chicago, IL; Taub Institute for Research on Alzheimer's Disease and the Aging Brain (L.S.H.), College of Physicians and Surgeons; Department of Neurology (L.S.H.), Columbia University, New York; Division of Neurology (G.-Y.R.H.), University of British Columbia, Vancouver, Canada; Department of Psychiatry and Human Behavior (E.D.H.), Alpert Medical School of Brown University, Providence, RI; Department of Neurology and Penn Frontotemporal Degeneration Center (D.J.I.), Perelman School of Medicine, University of Pennsylvania, Philadelphia; National Institute of Neurological Disorders and Stroke (J.Y.K., A.S.), National Institutes of Health, Bethesda, MD; Department of Neurology (J.C.M., B.P.), Houston Methodist, TX; Department of Psychiatry and Behavioral Sciences (C.U.O.), Johns Hopkins University, Baltimore, MD; Department of Neurology (P.S.P.), University of Colorado, Aurora; Cleveland Clinic Lou Ruvo Center for Brain Health (A.R., D.W.), Las Vegas, NV; Department of Neurology (E.D.R.), University of Alabama at Birmingham; Glenn Biggs Institute for Alzheimer's & Neurodegenerative Diseases (A.C.S.), UT Health San Antonio; Tanz Centre for Research in Neurodegenerative Diseases (M.C.T.), Division of Neurology, University of Toronto, Ontario, Canada; Department of Neurology (H.W.H., A.L.B., H.J.R.), Memory and Aging Center, University of California, San Francisco; Weill Institute for Neurosciences, San Francisco, CA; and Department of Neuroscience (R.R.), Mayo Clinic, Jacksonville, FL.

## Abstract

**Background and Objectives:**

*TMEM106B* has been proposed as a modifier of disease risk in FTLD-TDP, particularly in *GRN* pathogenic variant carriers. Furthermore, *TMEM106B* has been investigated as a disease modifier in the context of healthy aging and across multiple neurodegenerative diseases. The objective of this study was to evaluate and compare the effect of *TMEM106B* on gray matter volume and cognition in each of the common genetic FTD groups and in patients with sporadic FTD.

**Methods:**

Participants were enrolled through the ARTFL/LEFFTDS Longitudinal Frontotemporal Lobar Degeneration (ALLFTD) study, which includes symptomatic and presymptomatic individuals with a pathogenic variant in *C9orf72, GRN, MAPT, VCP, TBK1, TARDBP,* symptomatic nonpathogenic variant carriers, and noncarrier family controls. All participants were genotyped for the *TMEM106B* rs1990622 SNP. Cross-sectionally, linear mixed-effects models were fitted to assess an association between *TMEM106B* and genetic group interaction with each outcome measure (gray matter volume and UDS3-EF for cognition), adjusting for education, age, sex, and CDR+NACC-FTLD sum of boxes. Subsequently, associations between *TMEM106B* and each outcome measure were investigated within the genetic group. For longitudinal modeling, linear mixed-effects models with time by *TMEM106B* predictor interactions were fitted.

**Results:**

The minor allele of *TMEM106B* rs1990622, linked to a decreased risk of FTD, associated with greater gray matter volume in *GRN* pathogenic variant carriers under the recessive dosage model (N = 82, beta = 3.25, 95% CI [0.37–6.19], *p* = 0.034). This was most pronounced in the thalamus in the left hemisphere (beta = 0.03, 95% CI [0.01–0.06], *p* = 0.006), with a retained association when considering presymptomatic *GRN* pathogenic variant carriers only (N = 42, beta = 0.03, 95% CI [0.01–0.05], *p* = 0.003). The minor allele of *TMEM106B* rs1990622 also associated with greater cognitive scores among all *C9orf72* pathogenic variant carriers (N = 229, beta = 0.36, 95% CI [0.05–0.066], *p* = 0.021) and in presymptomatic *C9orf72* pathogenic variant carriers (N = 106, beta = 0.33, 95% CI [0.03–0.63], *p* = 0.036), under the recessive dosage model.

**Discussion:**

We identified associations of *TMEM106B* with gray matter volume and cognition in the presence of *GRN* and *C9orf72* pathogenic variants. The association of *TMEM106B* with outcomes of interest in presymptomatic *GRN* and *C9orf72* pathogenic variant carriers could additionally reflect TMEM106B's effect on divergent pathophysiologic changes before the appearance of clinical symptoms.

## Introduction

Frontotemporal lobar degeneration (FTLD) is one of the leading causes of dementia in individuals younger than 65 years and represents 10%–20% of all dementias. The term frontotemporal dementia (FTD) is used as an umbrella term for the spectrum of clinical manifestations that may result from FTLD, such as progressive changes in behavior or language difficulties. Some patients may also develop amyotrophic lateral sclerosis (ALS) or parkinsonism. One-third of patients show a strong family history, with most common genetic causes of FTD being autosomal dominant pathogenic variants in the progranulin (*GRN*) gene,^1,2^ the microtubule-associated protein tau (*MAPT*) gene,^3^ and the chromosome 9 open reading frame 72 (*C9orf72*) gene.^4^

Apart from autosomal dominant pathogenic variants causing FTD, additional genetic risk factors have been identified. In a genome-wide association study (GWAS) for the subgroup of patients with FTLD characterized by TDP-43 pathology (FTLD-TDP), *TMEM106B* was identified as a risk factor.^5^ The major allele (A) of the lead variant in the *TMEM106B* locus (rs1990622) was associated with an increased risk for developing FTLD-TDP or, alternatively, the minor allele (G) conferred protection. Of interest, the association with *TMEM106B* was most pronounced in the subset of patients with FTLD-TDP carrying a *GRN* pathogenic variant,^5^ implying that disease risk imposed by autosomal dominant pathogenic variants is also subject to genetic modifiers. In a GWAS of symptomatic *GRN* cases vs population controls, individuals carrying the minor *TMEM106B* haplotype indeed showed a 50% lower chance of developing disease symptoms as compared with *GRN* pathogenic variant carriers without the minor *TMEM106B* haplotype.^6^ Several other reports support the reduced disease penetrance associated with the minor (protective) *TMEM106B* haplotype,^7^ in particular in patients with *GRN* pathogenic variants.^8^ Strikingly, an obligate *GRN* pathogenic variant carrier was still unaffected in their 80s and found to be a homozygous carrier of the minor *TMEM106B* haplotype.^9^ This suggests that carrying 2 copies of the *TMEM106B* minor allele may counteract the disease-causing effects of the *GRN* pathogenic variant. A protective effect of the minor allele of *TMEM106B* rs1990622 SNP has also been demonstrated in *C9orf72* repeat expansion carriers, although less prominent compared with *GRN* pathogenic variant carriers.^10^ Although this has implications for genetic counselling, genotyping *TMEM106B* in *GRN* pathogenic variant carriers in the diagnostic setting is not routinely being performed.

*TMEM106B* has also been investigated as a disease modifier in the context of healthy aging. In elderly adults, the major risk allele of rs1990622 is associated with a smaller volume of the superior temporal gyrus, especially in the left hemisphere,^11^ with more advanced TDP-43 pathology at autopsy,^12^ increased biological aging in the prefrontal cortex,^13^ worse cognitive function,^13^ and decreased neuronal proportion.^14^ Moreover, in patients with FTD carrying 2 copies of the risk allele (AA) compared with the (AG+GG) group, lower cortical gray matter volumes in the frontal, temporal, cingulate, and insula cortices were noted.^15^ *TMEM106B* has also been shown to be a modulator of gray matter volume in presymptomatic pathogenic variant carriers^16^ and of cognitive trajectories over time among patients with clinical FTD.^17^ However, associations of *TMEM106B* with structural imaging and cognition within different FTD genetic groups remain to be investigated. Beyond FTLD, *TMEM106B* has been implicated in TDP-43 pathology in Alzheimer disease (AD),^18^ cognition in Parkinson disease (PD),^17^ and ALS, though with conflicting findings in directionality of effects in ALS.^19,20^

In this study, we aimed to investigate the modifying effects of *TMEM106B* in the largest collection of patients with systematically ascertained FTD and families from the ARTFL/LEFFTDS Longitudinal Frontotemporal Lobar Degeneration (ALLFTD) study, on gray matter volume and cognitive measures. Understanding the modifying effects of *TMEM106B* across genetic FTD subtypes is crucial in light of genetic counselling and the development of gene-based therapies.

## Methods

### Study Participants and Genetic Analysis

Participants were enrolled through Advancing Research and Treatment for Frontotemporal Lobar Degeneration (ARTFL, NCT02365922) and Longitudinal Evaluation of Familial Frontotemporal Dementia Subjects (LEFFTDS, NCT02372773)^21^ which combined into the ARTFL/LEFFTDS Longitudinal Frontotemporal Lobar Degeneration (ALLFTD, NCT04363684) study. These studies enrolled participants through a consortium of 27 centers across the United States and Canada between 2015 and 2023. Here, we report data from the most recent study visits for each participant as of October 26, 2023.

ALLFTD participants had genetic testing at the University of California, Los Angeles, using published methods.^22^ Briefly, DNA samples were screened for genes previously implicated in neurodegenerative diseases, including *GRN*, *MAPT, TBK1, VCP, TARDBP,* using targeted sequencing or whole-exome sequencing. The presence of hexanucleotide repeat expansions in *C9orf72* was detected using both fluorescent and repeat-primed PCR. *TMEM106B* rs1990622 genotyping was performed by real-time PCR on a LightCycler 480 System using Taqman SNP Genotyping Assays (#C__11171598_20). Assays were run in duplicate.

Genome-wide SNP genotyping data were used to perform lineage analysis using PLINK, as previously described.^23^ Briefly, QC was performed to remove individuals with low call rate and filter autosomal SNPs for missingness, frequency, and deviation from Hardy-Weinberg equilibrium. Genetic ancestry was inferred by projecting genotyped samples into the principal components of the 1000 Genomes reference panel, using R package bigsnpr. Identity-by-descent (IBD) estimates were then calculated to determine relatedness, followed by family-network identification and pedigree reconstruction using PRIMUS.^23^

Individuals with clinical data (clinical phenotype, age at visit) and genetic data (pathogenic variant in *C9orf72*, *GRN*, *MAPT*, *VCP*, *TBK1*, *TARDBP*, or noncarrier) available were retained. For affected nonpathogenic variant carriers, we only retained those with an FTD spectrum disorder, defined as either behavioral variant FTD (bvFTD), FTD with amyotrophic lateral sclerosis, corticobasal syndrome (CBS), progressive supranuclear palsy (PSP), agrammatic/nonfluent primary progressive aphasia, or semantic variant PPA.

### Data Collection of Outcome Measures

#### Neuroimaging Outcome: Gray Matter Volume

Image acquisition and processing were conducted as described previously.^24^ Before any preprocessing of the images, all T1-weighted images underwent quality control assessment at the Mayo Clinic Rochester in which images with excessive motion or other image artifacts were excluded. The images were processed by the UCSF Memory and Aging Center Imaging Core. The N3 algorithm was used for bias field correction of the T1-weighted images,^25^ and SPM12 (Wellcome Trust Center for Neuroimaging, London, UK,^26^ fil.ion.ucl.ac.uk/spm) unified segmentation for segmentation of the images.^27^ By nonlinear registration template generation using the Large Deformation Diffeomorphic Metric Mapping framework,^28^ a customized group template was generated from the segmented gray and white matter tissues and CSF. Participants' native space gray and white matter were geometrically normalized to the group template, modulated, and then smoothed in the group template. The applied smoothing used a Gaussian kernel with 8∼mm full width half maximum. Every step of the transformation was carefully inspected from the native space to the group template. From individual participants' smoothed, modulated gray matter in template space, regional volume estimates were calculated by taking the mean of all voxels in several a priori regions of interest (ROIs).^29^ The ROIs are summarized in eTable 1. All measures were expressed as a percentage of total intracranial volume.

#### Cognitive Outcome

Cognition was defined using the National Alzheimer's Coordinating Center Uniform Data Set (v3.0) executive function composite score (UDS3-EF), as described previously.^30,31^ The UDS3-EF is an item response theory-based composite derived from 7 total UDS3-EF test scores: category fluency (animals and vegetables; total correct), lexical fluency (F and L words; total correct), number span backward (total correct trials), and Trail Making Test parts A and B (correct lines per minute).^30,31^

### Neurofilament Light Chain Concentrations

Plasma neurofilament concentrations were determined as described previously.^32^ Neurofilament light chain (NfL) concentrations were quantified in duplicate using the ultrasensitive HDX analyzer by single-molecule array (Simoa) technology (Quanterix) by investigators blinded to clinical group allocation.^32^

### Statistical Analysis

All analyses were conducted in R (version 4.2.2). Linear mixed-effects analyses were conducted with the function ‘lmer’ in the R package ‘lme4’ (version 1.1.31).

For all cross-sectional analyses, the last available visit with the outcome measure available was used. Linear mixed models were fitted for the assessment of the main effect of the genetic groups according to their affection status (symptomatic/asymptomatic) on outcome variables, with individuals grouped by genetic status and affection status (eTable 2), with education, sex, age at visit, and CDR Dementia Staging Instrument plus Behavior and Language domains from the National Alzheimer's Disease Coordinating Center Frontotemporal Lobar Degeneration module (CDR+NACC-FTLD) sum of boxes score^33^ as fixed covariates and pedigree as a random effect. Owing to sample size limitations (<10), only nonpathogenic variant carriers and individuals with a pathogenic variant in *C9orf72*, *GRN*, or *MAPT* were considered.

To investigate the effect of the *TMEM106B* rs1990622 genotype on gray matter volume and cognition, linear mixed models were fitted with education, age, sex, genetic status, and the CDR+NACC-FTLD sum of boxes as covariates. The statistical analyses were performed under an additive (AA vs AG vs GG) and recessive [(AA+AG) vs GG] genetic model, where A and G are the major and minor allele, respectively. Secondary subgroup analyses were conducted in affected individuals only, a participant was defined as affected when the primary clinical phenotype was different from ‘clinically normal’.

In addition, the effect of *TMEM106B* genotype on gray matter volume and cognition was assessed in linear mixed-effects models with interaction testing between the *TMEM106B* genotype and genetic groups (noncarrier, *GRN*, *MAPT*, or *C9orf72*). If *p* < 0.05 for the interaction term TMEM106B*genetic group, linear mixed models were fitted for the individuals belonging to that genetic group, respectively (subgroup analyses), with education, age at visit, sex, and CDR+ NACC-FTLD sum of boxes as a covariate.

In longitudinal models, we used linear mixed-effects models with random slopes and intercepts [(time since baseline | participant ID) + (1 | pedigree ID)] to evaluate the association between *TMEM106B* genotype dosage and longitudinal changes in gray matter volume and cognition. Each participant's baseline was defined as the first study visit with available imaging and cognitive data. Only participants with at least 2 timepoints and with at least 1 visit with a clinical phenotype different from clinically normal were included. To determine whether *TMEM106B* genotype dosages were associated with rates of change in clinical outcomes, we examined the interaction between *TMEM106B* genotype dosage and time since baseline visit, adjusting for baseline age, sex, education, and baseline CDR+NACC-FTLD sum of boxes. In addition, each genetic group was analyzed in separate models.

For the analyses with the gray matter volumes as outcome, the primary analysis was conducted with the total gray matter volume as outcome. If *p* < 0.05 for the association of *TMEM106B* genotype with total gray matter volume, secondary analyses with the individual ROIs were conducted. Sensitivity analyses were conducted excluding individuals with non-European ancestry.

### Standard Protocol Approvals, Registrations, and Patient Consents

The ALLFTD study was approved through the Trial Innovation Network at Johns Hopkins University. Local ethics committees at each of the sites approved the study, and all participants provided written informed consent or assent with proxy consent.

### Data Availability

Deidentified human/patient clinical, demographic, imaging, and plasma NfL data are available from ALLFTD on request. Investigators are required to complete the Request Clinical Data form on the request portal^34^ and to review the data sharing and publication policy. Data that could identify a participant are not provided. Any additional information required to reanalyze the data reported in this paper is available from the lead contact and ALLFTD.

## Results

### Association of Genetic Group and Affection Status With Gray Matter Volume and Cognition

A total of 1,798 participants met the inclusion criteria for this study (Table 1). For gray matter volumetric measures, data were available for 958 participants (eTable 3). The UDS3-EF composite score was available for 1,581 participants (eTable 4).

**Table 1:** Demographic Characteristics for ALLFTD Participants (N = 1,798)

Characteristic	All pathogenic variant carriers	*C9orf72*+	*GRN*+	*MAPT*+	Noncarriers
Sample size	523	254	118	124	1,275
Age at visit (y), mean (SD)	53.95 (14.09)	53.74 (14.03)	59.36 (12.32)	48.73 (12.91)	62.82 (12.27)
Female, n (%)	293 (56.02)	146 (57.48)	61 (51.69)	72 (58.06)	618 (48.47)
Education (y), mean (SD)	15.48 (2.59) NA: 2	15.51 (2.50)	15.42 (2.97)	15.55 (2.44) NA: 1	16.05 (2.62)
Race, n					
EUR	501	249	110	119	1,159
Non-EUR	18	2	7	2	98
Unknown	3	3	1	0	18
*TMEM106B* rs1990622, n					
A/A	210	97	54	47	405
A/G	243	120	56	60	626
G/G	70	37	8	17	244
CDR®+NACC-FTLD Global, n					
0	209	109	43	47	279
0.5	74	38	13	19	187
≥1	221	94	60	54	766
Unknown	19	13	2	4	43
Primary clinical phenotype, n					
Clinically normal	210	110	44	48	284
MBI/MCI	46	23	9	13	57
bvFTD	174	76	38	51	334
ALS	12	12	0	0	0
FTD-ALS	17	14	0	0	20
PPA	17	5	9	1	242
CBS	15	2	10	1	138
PSP	4	2	0	2	200
Other	28	10	8	8	0
UDS3-EF (composite z-score) mean (SD)	−0.55 (1.46) NA: 90	−0.56 (1.41) NA: 30	−0.75 (1.50) NA: 33	−0.35 (1.57) NA: 18	−1.24 (1.39) NA: 228

Abbreviations: ALS = amyotrophic lateral sclerosis; bvFTD = behavioral variant frontotemporal dementia; CBS = corticobasal syndrome; CDR+NACC FTLD Global = CDR Dementia Staging Instrument plus Behavior and Language domains from the National Alzheimer's Disease Coordinating Center Frontotemporal Lobar Degeneration module global score; EUR = European; FTD = frontotemporal dementia; MBI/MCI = mild behavioral impairment/mild cognitive impairment; PPA = primary progressive aphasia; PSP = progressive supranuclear palsy; UDS3-EF = National Alzheimer's Coordinating Center Uniform Data Set (v3.0) executive function composite score.

First, we investigated the association between the gene-affection status (combined pathogenic variant and affection status) and our outcomes of interest: total gray matter volume and cognition (defined by UDS3-EF composite score), adjusting for education, age at visit, sex, and CDR+NACC-FTLD sum of boxes. As expected, being symptomatic, regardless of genetic status, was associated with lower total gray matter volumes and lower UDS3-EF scores (eTable 5). In addition, being a presymptomatic *C9orf72* pathogenic variant carrier was associated with lower total gray matter volumes (beta = −1.99, 95% CI [−2.80 to −1.19], *p* = 1.68 × 10^−6^) compared with clinically normal nonpathogenic variant carriers (eTable 5).

### Association of *TMEM106B* rs1990622 With Gray Matter Volume

Next, we investigated the association between *TMEM106B* rs1990622 and total gray matter volume in the complete cohort, including patients with sporadic and genetic FTD, presymptomatic carriers and nonpathogenic variant carrier controls. In linear mixed models with genetic status, years of education, sex, age at visit, and CDR+ NACC-FTLD sum of boxes score as fixed covariates and pedigree as a random effect, *TMEM106B* rs1990622 did not statistically associate with total gray matter volume with our sample sizes, neither in the additive dosage model nor in the recessive model (eTable 6). In subgroup analyses in all affected individuals, including sporadic and genetic FTD, *TMEM106B* rs1990622 did also not statistically associate with total gray matter volume (eTable 7) (*p* > 0.05).

Fitting the linear mixed-interaction model between *TMEM106B* rs1990622 and genetic group (nonpathogenic variant carrier, *GRN*, *MAPT* or *C9orf72*), with fixed covariates: years of education, sex, age at visit, and CDR+NACC-FTLD sum of boxes and with pedigree as a random effect, a protective effect of the minor allele of *TMEM106B* rs1990622 on total gray matter volume was observed with additive and recessive *TMEM106B* dosages in interaction analyses with *GRN* (Table 2). In both the additive and recessive models, statistically significant protective effects on the gray matter volumes of the right caudal anterior cingulate, right cerebellum, left rostral caudal anterior cingulate, and left frontal cortex were observed (Table 3). In the recessive model, the most significantly associated region was the left thalamus (*p* < 9.05 × 10^−5^, Table 3).

**Table 2:** Linear Mixed Model Statistics for *TMEM106* rs1990622 by Genetic Group Interaction on Total Gray Matter Volume

	Additive		Recessive	
	Coeff (95% CI)	*p* Value	Coeff (95% CI)	*p* Value
Education	0.09 (−0.001 to 0.17)	0.054	0.09 (−0.06 to 0.05)	0.052
Age at visit	−0.20 (−0.22 to −0.18)	< 2 × 10^−16^	−0.20 (−0.21 to −0.18)	< 2 × 10^−^^16^
Sex (female)	1.84 (1.42 to 2.27)	< 2 × 10^−16^	1.84 (1.40 to 2.25)	< 2 × 10^−^^16^
CDR®+NACC-FTLD SB	−0.45 (−0.50 to −0.41)	< 2 × 10^−16^	−0.46 (−0.51 to −0.41)	< 2 × 10^−^^16^
*GRN*	−1.92 (−2.99 to −0.86)	0.0004	−1.48 (−2.33 to −0.68)	0.0004
*C9orf72*	−2.11 (−2.95 to −1.26)	1.3 × 10^−6^	−2.27 (−2.94 to −1.66)	8.04 × 10^−^^12^
*MAPT*	−1.40 (−2.44 to −0.35)	0.009	−1.51 (−1.13 to −0.72)	0.0002
*TMEM106B*	−0.12 (−0.50 to 0.26)	0.55	−0.46 (−1.18 to 0.23)	0.201
TMEM106B*GRN	1.33 (0.05 to 2.60)	0.049	4.23 (0.95 to 7.67)	0.014
TMEM106B*C9orf72	−0.17 (−0.96 to 0.63)	0.604	0.03 (−1.43 to 1.64)	0.971
TMEM106B*MAPT	−0.07 (−1.09 to 0.94)	0.881	1.38 (−1.13 to 2.84)	0.182

Abbreviations: CDR+NACC-FTLD SB = CDR Dementia Staging Instrument plus Behavior and Language domains from the National Alzheimer's Disease Coordinating Center Frontotemporal Lobar Degeneration module sum of boxes score.

**Table 3:** Linear Mixed Model Statistics for *TMEM106B* rs1990622*GRN Interaction on Individual Gray Matter Regions

	Additive		Recessive	
	Coeff (95% CI)	*p* Value	Coeff (95% CI)	*p* Value
Right caudal anterior cingulate	0.006 (0.003 to 0.009)	0.0008	0.01 (0.0008 to 0.02)	0.033
Right caudate	0.009 (0.002 to 0.02)	0.009	0.02 (−0.003 to 0.03)	0.106
Left rostral anterior cingulate	0.006 (0.0008 to 0.01)	0.022	0.02 (0.003 to 0.03)	0.016
Left frontal cortex	0.13 (0.02 to 0.24)	0.022	0.40 (0.10 to 0.68)	0.008
Right posterior cingulate	0.005 (0.0005 to 0.009)	0.029	0.009 (−0.002 to 0.02)	0.107
Right cerebellum	0.09 (0.004 to 0.17)	0.040	0.29 (0.07 to 0.50)	0.009
Left caudate	0.007 (0.0002 to 0.01)	0.044	0.02 (−0.001 to 0.03)	0.073
Right frontal cortex	0.11 (−0.0007 to 0.23)	0.052	0.37 (0.08 to 0.67)	0.014
Left thalamus	0.008 (−0.0008 to 0.02)	0.075	0.04 (0.02 to 0.07)	9.05 × 10^−^^5^
Right thalamus	0.007 (−0.002 to 0.02)	0.120	0.04 (0.01 to 0.06)	0.002
Left cerebellum	0.06 (−0.01 to 0.14)	0.113	0.26 (0.06 to 0.46)	0.013
Left parietal cortex	0.06 (−0.001 to 0.12)	0.056	0.19 (0.03 to 0.35)	0.018
Left temporal cortex	0.03 (−0.05 to 0.11)	0.508	0.23 (0.02 to 0.45)	0.032

Results are depicted for regions with *p* < 0.05 for either the additive or recessive *TMEM106B* genotype dosage*GRN interaction.

In subgroup analyses in *GRN* pathogenic variant carriers, *TMEM106B* remained associated with the total gray matter volume in the recessive model (beta = 3.25, 95% CI [0.37–6.19], *p* = 0.034), with the left thalamic region as an individual region of interest with the highest association (beta = 0.03, 95% CI [0.01–0.060], *p* = 0.006) (eTable 8). Excluding the non-European *GRN* pathogenic variant carriers, *TMEM106B* remained associated with the total gray matter volume and left thalamic gray matter volume (beta = 3.44, 95% CI [0.72–6.23], *p* = 0.018 and beta = 0.03, 95% CI [0.01–0.06], *p* = 0.006, respectively).

**Figure 1:**
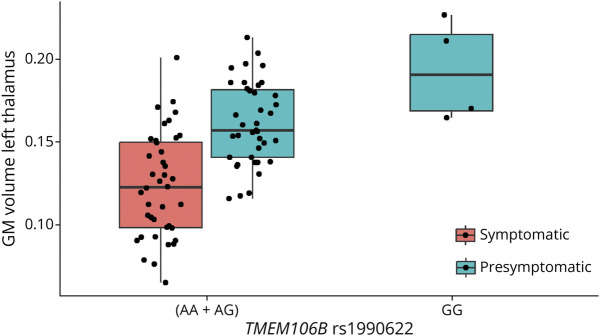
Left Thalamic Gray Matter Volume in *GRN* Pathogenic Variant Carriers, Grouped by Symptomatic Status and *TMEM106B* rs1990622 Genotype Dosages

*GRN* pathogenic variant carriers with the *TMEM106B* rs1990622*GG genotype are presymptomatic pathogenic variant carriers (Figure 1). Therefore, exploratory analyses were conducted that include only presymptomatic *GRN* pathogenic variant carriers. *TMEM106B* remained associated with the total gray matter volume (beta = 3.20, 95% CI [0.80–5.68], *p* = 0.016) and left thalamic gray matter volume (beta = 0.03, 95% CI [0.01–0.05], *p* = 0.003) in presymptomatic *GRN* pathogenic variant carriers in the recessive model after controlling for years of education, sex, and age at visit (eTable 9). Excluding the non-European *GRN* presymptomatic individual did not materially affect the findings with observed estimates of beta = 3.16, 95% CI [0.73–5.68], *p* = 0.018 and beta = 0.03, 95% CI [0.01–0.05], *p* = 0.003 for the total gray matter volume and left thalamic gray matter volume, respectively.

**Figure 2:**
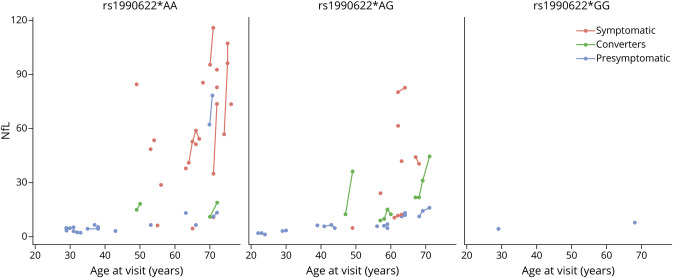
Scatter Plot Depicting the Age at Visit (X-Axis) and NfL Levels (Y-Axis) For All *GRN* Pathogenic Variant Carriers With Imaging Data and NfL Levels Measured, According to *TMEM106B* rs1990622 Genotype Blue dots: presymptomatic *GRN* pathogenic variant carriers, red dots: symptomatic *GRN* pathogenic variant carriers, green dots: *GRN* pathogenic variant carriers that converted from presymptomatic to symptomatic status. The lines connect data points that come from the same *GRN* pathogenic variant carrier.

The mean age of onset of the affected *GRN* pathogenic variant carriers in our total cohort with bvFTD, CBS, or PPA as primary diagnosis is 59.23 ± 9.23 years old. The presymptomatic *GRN* pathogenic variant carriers that carry the *TMEM106B* rs1990622*GG genotype are 29, 45, 49, and 68 years old at their last visit. NfL levels were available for the presymptomatic *GRN* pathogenic variant carriers with *TMEM106B* rs1990622*GG with an age at visit of 29 and 68 years old, respectively. Figure 2 depicts the age at visit and NfL levels for all *GRN* pathogenic variant carriers with NfL levels available at the time of imaging. Visually, it can be observed that the presymptomatic *GRN* pathogenic variant carrier with *TMEM106B* rs1990622*GG genotype with a current age of 68 years had among the lowest NfL levels (7.967 pg/mL), compared with both symptomatic (mean = 61.250 pg/mL) and presymptomatic *TMEM106B* rs1990622*AA and rs1990622*AG genotype *GRN* pathogenic variant carriers (mean = 24.774 pg/mL) within the same age range (65–77 years).

Longitudinally, the analyses were conducted with the additive model for *TMEM106B* rs1990622 in affected *GRN* pathogenic variant carriers. Statistical analyses were conducted for comparison of the rs1990622*AA group vs rs1990622*AG in affected *GRN* pathogenic variant carriers. We found no differences in the rate of decline in total gray matter volume across rs1990622*AG carriers vs rs1990622*AA carriers (beta = 0.536, 95% CI [−1.25 to 2.19], *p* = 0.526).

### Association of *TMEM106B* rs1990622 With Cognition

In linear mixed models with genetic status, years of education, sex, age at visit, and CDR+ NACC-FTLD sum of boxes score as fixed covariates and pedigree as a random effect, *TMEM106B* rs1990622 did not statistically associate with UDS3-EF across the complete cohort, neither in the additive dosage model nor in the recessive model (eTable 6), or in subgroup analyses in all affected individuals (eTable 7).

Fitting the linear mixed-interaction model between *TMEM106B* rs1990622 and genetic group (nonpathogenic variant carrier, *GRN*, *MAPT*, or *C9orf72*), with as fixed covariates years of education, sex, age at visit, and CDR+NACC-FTLD sum of boxes and with pedigree as a random effect, an effect of *TMEM106B* rs1990622 on UDS3-EF score in *C9orf72* pathogenic variant carriers was observed with recessive *TMEM106B* dosages (Table 4).

**Table 4:** Linear Mixed Model Statistics for *TMEM106B* rs1990622 by Genetic Group Interaction on UDS3-EF

	Additive		Recessive	
	Coeff (95% CI)	*p* Value	Coeff (95% CI)	*p* Value
Education	0.06 (0.04 to 0.08)	3.49 × 10^−11^	0.06 (0.04 to 0.08)	2.62 × 10^−^^11^
Age at visit	−0.03 (−0.04 to −0.03)	< 2 × 10^−16^	−0.03 (−0.04 to −0.03)	< 2 × 10^−^^16^
Sex (female)	−0.02 (−0.11 to 0.07)	0.696	−0.02 (−0.11 to 0.07)	0.690
CDR®+NACC-FTLD SB	−0.16 (−0.17 to −0.15)	< 2 × 10^−16^	−0.16 (−0.17 to −0.15)	< 2 × 10^−^^16^
*GRN*	−0.07 (−0.34 to 0.20)	0.591	0.03 (−0.17 to 0.23)	0.781
*C9orf72*	0.02 (−0.18 to 0.22)	0.834	0.09 (−0.06 to 0.23)	0.239
*MAPT*	0.14 (−0.13 to 0.41)	0.317	0.09 (−0.11 to 0.30)	0.380
*TMEM106B*	−0.07 (−0.15 to 0.004)	0.064	−0.12 (−0.26,0.01)	0.070
TMEM106B*GRN	0.24 (−0.06 to 0.54)	0.114	0.75 (−0.01 to 1.51)	0.052
TMEM106B*C9orf72	0.16 (−0.03 to 0.35)	0.096	0.42 (0.05 to 0.79)	0.026
TMEM106B*MAPT	−0.09 (−0.35 to 0.17)	0.487	−0.11 (−0.60 to 0.39)	0.673

Abbreviations: CDR+NACC-FTLD SB = CDR Dementia Staging Instrument plus Behavior and Language domains from the National Alzheimer's Disease Coordinating Center Frontotemporal Lobar Degeneration module sum of boxes score.

In subgroup analyses in *C9orf72* pathogenic variant carriers, *TMEM106B* remained associated with UDS3-EF in the recessive model (beta = 0.36, 95% CI [0.05–0.66], *p* = 0.021), and in subgroup analyses in presymptomatic *C9orf72* pathogenic variant carriers (beta = 0.33, 95% CI [0.03–0.63], *p* = 0.036). Similar estimates were obtained on conducting sensitivity analyses in *C9orf72* pathogenic variant carriers of European ancestry only (beta = 0.40, 95% CI [0.09–0.70], *p* = 0.011) and presymptomatic *C9orf72* pathogenic variant carriers of European ancestry only (beta = 0.40, 95% CI [0.10–0.71], *p =* 0.011). In symptomatic *C9orf72* pathogenic variant carriers, there was no effect of *TMEM106B* on UDS3-EF (beta = 0.31, 95% CI [−0.19 to 0.81], *p* = 0.232).

We did not identify statistically significant longitudinal trajectory differences according to *TMEM106B* genotype group (data not shown). In presymptomatic *C9orf72* pathogenic variant carriers with at least 2 visits, there was no significant decline in cognitive trajectory over time. However, taking into account all the longitudinally collected visits in presymptomatic *C9orf72* pathogenic variant carriers, we found in both the additive (beta = 0.22, 95% CI [0.05–0.39], *p* = 0.014) and recessive (beta = 0.45, 95% CI [0.13–0.78], *p* = 0.008) model (eTable 10), that the minor allele of *TMEM106B* rs1990622 is associated with an increased UDS3-EF score, in line with the cross-sectional data taking only the last visit into account.

## Discussion

*TMEM106B* was initially identified as genetic risk factor for FTLD-TDP. Since then, it has been shown to not only act as a modifier of disease penetrance in FTLD-TDP but also as a modifier of pathologic, imaging, and clinical characteristics of FTD and related neurodegenerative diseases. To further confirm the association of *TMEM106B* SNPs with imaging and clinical characteristics in FTD and to evaluate its role in the different genetic groups of autosomal dominant FTD, we performed association analyses in the largest available systematically ascertained cohort of patients with FTD.

In our complete cohort with imaging data available, no significant association of gray matter brain volumes with *TMEM106B* was detected. However, in *GRN* pathogenic variant carriers, carrying 2 copies of the minor allele of *TMEM106B* was associated with a larger total gray matter volume. This was most pronounced in the thalamus in the left hemisphere, a finding that remained in a subgroup of presymptomatic *GRN* pathogenic variant carriers. Thalamic atrophy is a common feature in frontotemporal dementia, and especially in *GRN* pathogenic variant carriers, asymmetry in thalamic volumes is apparent.^35^ Furthermore, *GRN* presymptomatic pathogenic variant carriers display changes in intrinsic connectivity networks, with the thalamus as a key hub.^36^ This is in line with findings in mice with homozygous *GRN* deletions (*GRN*^−/−^),^37^ where microglial activation in the ventral thalamus drives neurodegeneration in the thalamocortical circuit.^37^ Of interest, patients with FTLD-*GRN* and *GRN*^−/−^ mice show similar transcriptomic and histopathologic changes in the thalamus, not only in microglia but also in astrocytes, promoting neurodegeneration.^38^ Other regions that appear altered in response to *TMEM106B* are the frontal, temporal, parietal, anterior cingulate areas, insula, and cerebellum, in line with known patterns of atrophy described in *GRN* pathogenic variant carriers^39^ and in patients with FTLD-TDP type A, the pathology uniformly present in patients with *GRN* pathogenic variants. In addition, previous research showed an effect of *TMEM106B* in these regions in a clinically diagnosed FTD cohort.^15^

Importantly, the GRN pathogenic variant carriers with 2 copies of the minor allele of TMEM106B were all presymptomatic at time of imaging. With a mean age of onset of 59 years in affected *GRN* pathogenic variant carriers in our total cohort, it cannot be excluded that these presymptomatic *GRN* pathogenic variant carriers will still develop FTD at a later age; however, one of these presymptomatic *GRN* pathogenic variant carriers remained without symptoms at 68 years of age. The strikingly low NfL level of this participant compared with *GRN* pathogenic variant carriers within the same age range (65–77 years), also well below the mean value of phenoconverters,^40^ supports the hypothesis that carrying 2 copies of the minor allele of *TMEM106B* might offer protection against developing FTD, or at a minimum a delay in disease onset.

In *C9orf72*, we did not observe an association between *TMEM106B* and (sub)cortical atrophy. In fact, at the presymptomatic stage, we found that irrespective of the *TMEM106B* genotype, the presence of *C9orf72* is associated with lower gray matter volumes in comparison with clinically normal nonpathogenic variant carriers, consistent with prior work showing structural brain changes occurring 10–40 years before onset.^39^ In *GRN* pathogenic variant carriers, on the other hand, changes in brain volume occur only within a few years proximity to onset of symptomatic FTD.^24,41^ Moreover, although the rate of volume loss differs between *C9orf72* and *GRN,* with an attenuated atrophy rate after onset of symptomatic FTD in *C9orf72* and with an acceleration of atrophy rate after onset in *GRN,* their rate of functional decline is similar.^41^ Hence, there might be earlier and divergent pathophysiologic changes in *C9orf72* as compared with *GRN* pathogenic variant carriers in the presymptomatic phase, with the early loss of gray matter volume in *C9orf72* pathogenic variant carriers masking a potential effect of *TMEM106B*.

In contrast to structural imaging, we did identify a protective effect of the *TMEM106B* rs1990622 minor allele on cognition in *C9orf72*, especially in presymptomatic *C9orf72* pathogenic variant carriers. With participants with a *C9orf72* repeat expansion already showing signs of neurodegeneration (e.g., gray matter loss) before symptom onset, we hypothesize that *TMEM106B* is able to modulate the resilience against developing clinical FTD during these early stages of disease. In support of this hypothesis, homozygosity for the minor allele has been shown to protect *C9orf72* carriers from developing FTD but not from developing ALS.^10^ Moreover, discordance between the presence of disease pathology and effects on cognition in the aging population is a known phenomenon, and *TMEM106B* has been suggested as a potential modifier of this “cognitive resilience,” with the minor allele of *TMEM106B* rs1990622 being associated with a better performance than expected based on pathologic burden.^42^

Previous studies focusing on presymptomatic genetic FTD have identified modulating effects of *TMEM106B* genotype on gray matter volume in pathogenic variant carriers (combining *GRN, C9orf72, MAPT*) vs noncarrier family controls.^16^ It is important that a different distribution in genetic groups between our study and the previously conducted studies^16^ can be noted, with *GRN* being the largest group and *MAPT* being the smallest group in the previous studies, while in this study, the pathogenic variant carriers are enriched for *C9orf72* and *MAPT* carriers, with *GRN* being the smallest group (22% vs 56% in the previous studies). Furthermore, in this study, we also included a sporadic FTD cohort without pathogenic variants identified in the known FTD genes. Hence, we investigated the association of *TMEM106B* with gray matter volume and cognition in each genetic group separately through interaction modelling and subgroup analyses. We identified associations of *TMEM106B* in the *GRN* and *C9orf72* genetic groups. This is in line with *TMEM106B* being identified as a modifier in those with TDP-43 pathology^5,6^ but not in most other clinical FTD cohorts of non-TDP^43^ or unknown pathology,^5^ with a few exceptions^7,15^ potentially due to a substantial proportion of cases with FTLD-TDP pathology.^7,15^ Beyond FTLD-TDP, *TMEM106B* is associated with hippocampal sclerosis of aging,^18^ with or without accompanying Alzheimer type pathology, with hippocampal sclerosis in Lewy body disease,^44^ and with limbic-predominant age-related TDP-43 proteinopathy (LATE-NC),^45^ all characterized by the presence of TDP-43 proteinopathy. Furthermore, TDP-43 inclusions are also present in Alzheimer disease and Parkinson disease,^46^ explaining the broader modifying roles of *TMEM106B* in endophenotypes such as cognition across neurodegenerative diseases.

Strikingly, TMEM106B filaments form aggregates in the brain in elderly and across neurodegenerative diseases,^47^ with the risk allele associated with greater fibril formation^48^ and enhanced TDP-43 dysfunction.^49^ Although fibril accumulation has been found to be a common age-related phenomenon, fibril aggregates were especially abundant in patients with *GRN* pathogenic variants.^50^ Both progranulin and TMEM106B are important players in lysosomal health.^47^ TMEM106B is a transmembrane glycoprotein that primarily localizes to lysosomal membranes where it is proteolytically processed. Progranulin is cleaved in the lysosome into functional granulins, and homozygous loss-of-function pathogenic variants in *GRN* cause the lysosomal storage disorder neuronal ceroid lipofuscinosis 11. In addition to convergence of pathomechanisms between *GRN* and *TMEM106B*, TMEM106B-induced lysosomal defects due to increased TMEM106B expression have been shown to be C9orf72-dependent.^51^ Altogether, these studies support a specific role for *TMEM106B* as a modifier in FTLD-TDP pathophysiology.

We acknowledge that there are limitations with this work. The UDS3-EF endpoint is composed of tests heavily loaded on dorsolateral frontal function, which may lead to an underestimation of cognitive impairment, in particular in participants with PPA. However, executive functioning is affected in all FTD genetic groups and clinical phenotypes,^52^ and it is shown that processing speed and executive functioning deficits seem early in presymptomatic familial FTD.^39^ Although we investigated modifying effects of *TMEM106B* in the largest collection of patients with systematically ascertained FTD and families from the ALLFTD study, generalization of our findings may be hampered as individuals in this study presented with high educational levels (mean of 15.88 years). Furthermore, the number of individuals with a *GRN* pathogenic variant and 2 copies of the minor (protective) allele of *TMEM106B* is small. This supports a role for *TMEM106B* in reducing disease penetrance but consequently also leads to an underrepresentation of *GRN* pathogenic variant carriers homozygous for the minor *TMEM106B* allele in research studies. Therefore, extensive recruitment of unaffected family members of *GRN* pathogenic variant carriers followed by genetic analyses of *TMEM106B* and *GRN* will be required to specifically identify those individuals who carry a *GRN* pathogenic variant and 2 copies of the *TMEM106B* minor allele to validate our findings. In addition, to reach the maximum sample size for each outcome measure of interest, the last visit with the measure of interest available was selected. In this way, the analyses differ in their set of unique individuals and their respective time point of assessment, precluding multivariate analysis of variance studies to assess simultaneously associations between *TMEM106B,* imaging, and cognition in the same cohort. Although we used the largest data set possible, some of our negative statistical associations may be due to small sample sizes. Despite these limitations, we confirmed *TMEM106B* as a modifier in *GRN* and *C9orf72* pathogenic variant carriers, and reported distinct effects in different genetic groups. Importantly, we showed that *TMEM106B* already exerts effects in the presymptomatic stage of disease. With clinical trials ongoing for gene-based therapies for *GRN* and *C9orf72* pathogenic variant carriers, it is important to take *TMEM106B* genetic status into account in the clinical trial design and recruitment of participants.

## Disclosure

M. Vandebergh received funding for this project from the Queen Elisabeth Medical Foundation of Neurosciences (GSKE). E.M. Ramos receives research support from the NIH. N. Corriveau-Lecavalier reports no disclosures relevant to the manuscript. V.K. Ramanan has received research funding from the NIH and the Mangurian Foundation for Lewy Body disease research, has provided educational content for Medscape, has received speaker and conference session honoraria from the American Academy of Neurology Institute, is co-PI for a clinical trial supported by the Alzheimer's Association, is site Co-I for the Alzheimer's Clinical Trials Consortium, and is a site clinician for clinical trials supported by Eisai, the Alzheimer's Treatment and Research Institute at USC, and Transposon Therapeutics Inc. J. Kornak has provided expert witness testimony for Teva Pharmaceuticals in Forest Laboratories Inc. et al. v. Teva Pharmaceuticals USA, Inc., case numbers 1:14-cv-00121 and 1:14-cv-00686 (D. Del. filed 31 January 2014 and 30 May 2014 regarding the drug Memantine); and for Apotex/HEC/Ezra in Novartis AG et al. v. Apotex Inc., case number 1:15-cv-975 (D. Del. filed 26 October 2015 regarding the drug Fingolimod); he has also given testimony on behalf of Puma Biotechnology in Hsingching Hsu et al. vs Puma Biotechnology Inc. et al. 2018 regarding the drug Neratinib; and he receives research support from the NIH. C. Mester, T. Kolander, and D. Brushaber report no disclosures relevant to the manuscript. A.M. Staffaroni received research support from the NIA/NIH, the Bluefield Project to Cure FTD, the Association for Frontotemporal Dementia, the ALS Association, the Rainwater Charitable Foundation, and the Larry L. Hillblom Foundation, has provided consultation to Alector, Lilly/Prevail Therapeutics, Passage Bio, and Takeda, and serves on the scientific review board for ADDF. D. Geschwind and A. Wolf report no disclosures relevant to the manuscript. K. Kantarci served on the Data Safety Monitoring Board for Takeda Global Research & Development Center and data monitoring boards of Pfizer and Janssen Alzheimer Immunotherapy, and received research support from Avid Radiopharmaceuticals, Eli Lilly, the Alzheimer's Drug Discovery Foundation, and the NIH. T.F. Gendron and L. Petrucelli receive research support from the NIH. M. Van den Broeck, S. Wynants, and M.C. Baker report no disclosures relevant to the manuscript. S. Borrego-Écija is a recipient of the Joan Rodés Josep Baselga grant from the FBBVA. B. Appleby receives research support from the Centers for Disease Control and Prevention, the NIH, Ionis, Alector, and the CJD Foundation, and has provided consultation to Acadia, Ionis, and Sangamo. S. Barmada, A. Bozoki, D. Clark, and R. Ryan Darby report no disclosures relevant to the manuscript. B.C. Dickerson is a consultant for Acadia, Alector, Arkuda, Biogen, Denali, Eisai, Genentech, Lilly, Merck, Novartis, Takeda, and Wave Lifesciences, receives royalties from Cambridge University Press, Elsevier, and Oxford University Press, and receives grant funding from the NIA, the National Institute of Neurological Disorders and Stroke, the National Institute of Mental Health, and the Bluefield Foundation. K. Domoto-Reilly receives research support from the NIH and serves as an investigator for a clinical trial sponsored by Lawson Health Research Institute. J.A. Fields receives research support from the NIH. D.R. Galasko reports no disclosures relevant to the manuscript. N. Ghoshal has participated or is currently participating in clinical trials of anti-dementia drugs sponsored by Bristol Myers Squibb, Eli Lilly/Avid Radiopharmaceuticals, Janssen Immunotherapy, Novartis, Pfizer, Wyeth, SNIFF (The Study of Nasal Insulin to Fight Forgetfulness), and the A4 (Anti-Amyloid Treatment in Asymptomatic Alzheimer's Disease) trial, receives research support from the Tau Consortium and the Association for Frontotemporal Dementia, and is funded by the NIH. N. Graff-Radford receives royalties from UpToDate, has participated in multicenter therapy studies sponsored by Biogen, TauRx, and Lilly, and receives research support from the NIH. I.M. Grant reports no disclosures relevant to the manuscript. L.S. Honig receives research funding from Abbvie, Acumen, Alector, Biogen, BMS, Eisai, Genentech/Roche, Janssen/J&J, Transposon, UCB, and Vaccinex, and consulting fees from Biogen, Cortexyme, Eisai, Medscape, and Prevail/Lilly. G.-Y.R. Hsiung has served as an investigator for clinical trials sponsored by AstraZeneca, Eli Lilly, and Roche/Genentech, and receives research support from the Canadian Institutes of Health Research and the Alzheimer Society of British Columbia. E.D. Huey receives research support from the NIH. D. Irwin receives support from the NIH, the BrightFocus Foundation, and the Penn Institute on Aging. D.S. Knopman serves on the data and safety monitoring board of the DIAN-TU study, is a site principal investigator for clinical trials sponsored by Biogen, Lilly, and the University of Southern California, and is funded by the NIH. J. Kwan and G.C. Léger report no disclosures relevant to the manuscript. I. Litvan is supported by NIH grants: 2R01AG038791-06A, U01NS100610, U01NS80818, R25NS098999, U19 AG063911-1, and 1R21NS114764-01A1, and by the Michael J. Fox Foundation, the Parkinson Foundation, the Lewy Body Association, CurePSP, Roche, Abbvie, Biogen, Centogene, EIP-Pharma, Biohaven Pharmaceuticals, Novartis, Brain Neurotherapy Bio, and United Biopharma SRL—UCB; is a scientific advisor for Amydis and the Rossy Center for Progressive Supranuclear Palsy University of Toronto, and receives her salary from the University of California San Diego and as a chief editor of *Frontiers in Neurology*. J.S. Masdeu reports no disclosures relevant to the manuscript. M.F. Mendez receives research support from the NIH. C.U. Onyike receives research funding from the NIH, Lawton Health Research Institute, the National Ataxia Foundation, Alector, and Transposon, is supported by the Robert and Nancy Hall Brain Research Fund, and the Jane Tanger Black Fund for Young-Onset Dementias, and by a gift from Joseph Trovato, and is a consultant with Alector Inc., Acadia Pharmaceuticals, and Reata Pharmaceuticals. B. Pascual, P. Pressman, and A. Ritter report no disclosures relevant to the manuscript. E.D. Roberson has received research support from the NIH, the Bluefield Project to Cure Frontotemporal Dementia, the Alzheimer's Association, the Alzheimer's Drug Discovery Foundation, the BrightFocus Foundation, and Alector, has served as a consultant for AGTC and on a data monitoring committee for Lilly, and owns intellectual property related to tau and progranulin. A. Snyder and A. Campbell Sullivan report no disclosures relevant to the manuscript. M.C. Tartaglia has served as an investigator for clinical trials sponsored by Biogen, Avanex, Green Valley, Roche/Genentech, Bristol Myers Squibb, Eli Lilly/Avid Radiopharmaceuticals, and Janssen, and receives research support from the Canadian Institutes of Health Research. D. Wint, H.W. Heuer, and L.K. Forsberg report no disclosures relevant to the manuscript. A.L. Boxer receives research support from the NIH, the Tau Research Consortium, the Association for Frontotemporal Degeneration, the Bluefield Project to Cure Frontotemporal Dementia, Corticobasal Degeneration Solutions, the Alzheimer's Drug Discovery Foundation, and the Alzheimer's Association, has served as a consultant for Aeovian, AGTC, Alector, Arkuda, Arvinas, Boehringer Ingelheim, Denali, GSK, Life Edit, Humana, Oligomerix, Oscotec, Roche, TrueBinding, Wave, and Merck, and received research support from Biogen, Eisai, and Regeneron. H.J. Rosen has received research support from Biogen Pharmaceuticals, has consulting agreements with Wave Neuroscience, Ionis Pharmaceuticals, Eisai Pharmaceuticals, and Genentech, and receives research support from the NIH and the state of California. B.F. Boeve has served as an investigator for clinical trials sponsored by Alector, Biogen, Transposon, and Cognition Therapeutics, serves on the Scientific Advisory Board of the Tau Consortium which is funded by the Rainwater Charitable Foundation, and receives research support from the NIH. R.R. receives research funding from the NIH and the Bluefield Project to Cure Frontotemporal Dementia, is on the scientific advisory board of Arkuda Therapeutics, receives royalties from a progranulin-related patent, and is on the scientific advisory board of the Fondation Alzheimer. Go to Neurology.org/N for full disclosures.

## Appendix 1 Authors

**Table:**

Name	Location	Contribution
Marijne Vandebergh, PhD	VIB Center for Molecular Neurology; Department of Biomedical Sciences, University of Antwerp, Belgium	Drafting/revision of the manuscript for content, including medical writing for content; study concept or design; analysis or interpretation of data
Eliana Marisa Ramos, PhD	Department of Neurology, David Geffen School of Medicine, University of California, Los Angeles	Drafting/revision of the manuscript for content, including medical writing for content; major role in the acquisition of data; study concept or design; analysis or interpretation of data
Nick Corriveau-Lecavalier, PhD	Department of Neurology; Department of Psychiatry and Psychology, Mayo Clinic, Rochester, MN	Drafting/revision of the manuscript for content, including medical writing for content; analysis or interpretation of data
Vijay K. Ramanan, MD, PhD	Department of Neurology, Mayo Clinic, Rochester, MN	Drafting/revision of the manuscript for content, including medical writing for content; analysis or interpretation of data
John Kornak, PhD	Department of Epidemiology and Biostatistics, University of California, San Francisco	Drafting/revision of the manuscript for content, including medical writing for content; analysis or interpretation of data
Carly Mester, BA	Department of Quantitative Health Sciences, Mayo Clinic, Rochester, MN	Major role in the acquisition of data
Tyler Kolander, BA	Department of Neurology, Mayo Clinic, Rochester, MN	Major role in the acquisition of data
Danielle Elise Brushaber, BS	Department of Quantitative Health Sciences, Mayo Clinic, Rochester, MN	Major role in the acquisition of data
Adam M Staffaroni, PhD	Department of Neurology, Memory and Aging Center, University of California, San Francisco; Weill Institute for Neurosciences, San Francisco, CA	Drafting/revision of the manuscript for content, including medical writing for content; major role in the acquisition of data
Daniel H. Geschwind, MD, PhD	Institute for Precision Health, Departments of Neurology, Psychiatry and Human Genetics at David Geffen School of Medicine, UCLA	Major role in the acquisition of data
Amy A Wolf, BS	Department of Neurology, Memory and Aging Center, University of California, San Francisco; Weill Institute for Neurosciences, San Francisco, CA	Major role in the acquisition of data
Kejal Kantarci, MD	Department of Neurology, Mayo Clinic, Rochester, MN	Major role in the acquisition of data
Tania Gendron, PhD	Department of Neuroscience, Mayo Clinic, Jacksonville, FL	Major role in the acquisition of data
Leonard Petrucelli, PhD	Department of Neuroscience, Mayo Clinic, Jacksonville, FL	Major role in the acquisition of data
Marleen Van den Broeck, BS	Department of Biomedical Sciences, University of Antwerp; VIB Center for Molecular Neurology, VIB, Antwerp, Belgium	Major role in the acquisition of data
Sarah Wynants, BS	Department of Biomedical Sciences, University of Antwerp; VIB Center for Molecular Neurology, VIB, Antwerp, Belgium	Major role in the acquisition of data
Matthew Baker, BS	Department of Neuroscience, Mayo Clinic, Jacksonville, FL	Major role in the acquisition of data
Sergi Borrego-Écija, MD, PhD	Alzheimer's Disease and Other Cognitive Disorders Unit, Neurology Service, Hospital Clínic de Barcelona, Institut d'Investigacions Biomèdiques August Pi i Sunyer (IDIBAPS), Fundació Clínic per a la Recerca Biomèdica, Uni;	Drafting/revision of the manuscript for content, including medical writing for content; analysis or interpretation of data
Brian Appleby, MD	Department of Neurology, Case Western Reserve University, Cleveland, OH	Major role in the acquisition of data; analysis or interpretation of data
Sami Barmada, MD, PhD	Department of Neurology, University of Michigan, Ann Arbor	Major role in the acquisition of data
Andrea C. Bozoki, MD	Department of Neurology, University of North Carolina, Chapel Hill	Major role in the acquisition of data
David Clark, MD	Department of Neurology, Indiana University, Indianapolis	Major role in the acquisition of data
R. Ryan Darby, MD	Department of Neurology, Vanderbilt University, Nashville, TN	Major role in the acquisition of data
Bradford Clark Dickerson, MD	Department of Neurology, Case Western Reserve University, Cleveland, OH	Drafting/revision of the manuscript for content, including medical writing for content; major role in the acquisition of data; study concept or design; analysis or interpretation of data
Kimiko Domoto-Reilly, MD	Department of Neurology, University of Washington, Seattle	Major role in the acquisition of data
Julie A Fields, PhD	Department of Psychiatry and Psychology, Mayo Clinic, Rochester, MN	Major role in the acquisition of data
Douglas Galasko, MD	Department of Neurosciences, University of California, San Diego, La Jolla	Major role in the acquisition of data
Nupur Ghoshal, MD, PhD	Departments of Neurology and Psychiatry, Washington University School of Medicine, Washington University, St. Louis, MO	Drafting/revision of the manuscript for content, including medical writing for content; major role in the acquisition of data
Neill R. Graff-Radford, MD	Department of Neuroscience, Mayo Clinic, Jacksonville, FL,	Drafting/revision of the manuscript for content, including medical writing for content; major role in the acquisition of data
Ian M. Grant, MD, MA	Department of Psychiatry and Behavioral Sciences, Northwestern Feinberg School of Medicine, Chicago, IL	Major role in the acquisition of data
Lawrence S. Honig, MD, PhD	Taub Institute for Research on Alzheimer's Disease and the Aging Brain, College of Physicians and Surgeons; Department of Neurology, Columbia University, New York	Major role in the acquisition of data
Ging-Yuek R. Hsiung, MD, MHSc	Division of Neurology, University of British Columbia, Vancouver, Canada	Major role in the acquisition of data
Edward D. Huey, MD	Department of Psychiatry and Human Behavior, Alpert Medical School of Brown University, Providence, RI	Drafting/revision of the manuscript for content, including medical writing for content; major role in the acquisition of data
David John Irwin, MD	Department of Neurology and Penn Frontotemporal Degeneration Center, Perelman School of Medicine, University of Pennsylvania, Philadelphia	Drafting/revision of the manuscript for content, including medical writing for content; major role in the acquisition of data
David S. Knopman, MD	Department of Psychiatry and Psychology, Mayo Clinic, Rochester, MN	Drafting/revision of the manuscript for content, including medical writing for content; major role in the acquisition of data
Justin Y. Kwan, MD	National Institute of Neurological Disorders and Stroke, National Institutes of Health, Bethesda, MD	Drafting/revision of the manuscript for content, including medical writing for content; major role in the acquisition of data
Gabriel C. Léger, MD	Department of Neurosciences, University of California, San Diego, La Jolla	Drafting/revision of the manuscript for content, including medical writing for content; major role in the acquisition of data
Irene Litvan, MD	Department of Neurosciences, University of California, San Diego, La Jolla	Drafting/revision of the manuscript for content, including medical writing for content; major role in the acquisition of data
Joseph C. Masdeu, MD, PhD	Department of Neurology, Houston Methodist, TX	Major role in the acquisition of data
Mario F. Mendez, MD, PhD	Department of Neurology, David Geffen School of Medicine, University of California, Los Angeles	Major role in the acquisition of data
Chiadi U. Onyike, MD, MHS	Department of Psychiatry and Behavioral Sciences, Johns Hopkins University, Baltimore, MD	Major role in the acquisition of data
Belen Pascual, PhD	Department of Neurology, Houston Methodist, TX	Major role in the acquisition of data
Peter S. Pressman, MD	Department of Neurology, University of Colorado, Aurora	Drafting/revision of the manuscript for content, including medical writing for content; major role in the acquisition of data
Aaron Ritter, MD	Cleveland Clinic Lou Ruvo Center for Brain Health, Las Vegas, NV	Drafting/revision of the manuscript for content, including medical writing for content; major role in the acquisition of data
Erik D. Roberson, MD, PhD	Department of Neurology, University of Alabama at Birmingham	Drafting/revision of the manuscript for content, including medical writing for content; major role in the acquisition of data
Allison Snyder, MD	National Institute of Neurological Disorders and Stroke, National Institutes of Health, Bethesda, MD	Drafting/revision of the manuscript for content, including medical writing for content; major role in the acquisition of data
Anna Campbell Sullivan, PsyD	Glenn Biggs Institute for Alzheimer's & Neurodegenerative Diseases, UT Health San Antonio	Drafting/revision of the manuscript for content, including medical writing for content; major role in the acquisition of data
Maria Carmela Tartaglia, MD	Tanz Centre for Research in Neurodegenerative Diseases, Division of Neurology, University of Toronto, Toronto, Canada	Major role in the acquisition of data
Dylan Wint, MD	Cleveland Clinic Lou Ruvo Center for Brain Health, Las Vegas, NV	Major role in the acquisition of data
Hilary W. Heuer, PhD	Department of Neurology, Memory and Aging Center, University of California, San Francisco; Weill Institute for Neurosciences, San Francisco, CA	Drafting/revision of the manuscript for content, including medical writing for content; major role in the acquisition of data
Leah K. Forsberg, PhD	Department of Psychiatry and Psychology, Mayo Clinic, Rochester, MN	Drafting/revision of the manuscript for content, including medical writing for content; major role in the acquisition of data
Adam L. Boxer, MD, PhD	Department of Neurology, Memory and Aging Center, University of California, San Francisco; Weill Institute for Neurosciences, San Francisco, CA	Drafting/revision of the manuscript for content, including medical writing for content; major role in the acquisition of data
Howard J. Rosen, MD	Department of Neurology, Memory and Aging Center, University of California, San Francisco; Weill Institute for Neurosciences, San Francisco, CA	Drafting/revision of the manuscript for content, including medical writing for content; major role in the acquisition of data
Bradley F. Boeve, MD	Department of Neurology, Mayo Clinic, Rochester, MN	Drafting/revision of the manuscript for content, including medical writing for content; major role in the acquisition of data
Rosa Rademakers, PhD	VIB Center for Molecular Neurology; Department of Biomedical Sciences, University of Antwerp, Belgium; Department of Neuroscience, Mayo Clinic, Jacksonville, FL	Drafting/revision of the manuscript for content, including medical writing for content; major role in the acquisition of data; study concept or design; analysis or interpretation of data

## Appendix 2 Coinvestigators

**Table:**

Coinvestigators are listed at Neurology.org/N.

## Glossary

AD: Alzheimer disease
ALS: amyotrophic lateral sclerosis
CBS: corticobasal syndrome
FTD: frontotemporal dementia
FTLD: frontotemporal lobar degeneration
GWAS: genome-wide association study
PD: Parkinson disease
ROI: region of interest

## References

[1] Baker M, Mackenzie IR, Pickering-Brown SM, et al. Mutations in progranulin cause tau-negative frontotemporal dementia linked to chromosome 17. Nature. 2006;442(7105):916-919. doi:10.1038/nature05016 16862116

[2] Cruts M, Gijselinck I, van der Zee J, et al. Null mutations in progranulin cause ubiquitin-positive frontotemporal dementia linked to chromosome 17q21. Nature. 2006;442(7105):920-924. doi:10.1038/nature05017 16862115

[3] Hutton M, Lendon CL, Rizzu P, et al. Association of missense and 5'-splice-site mutations in tau with the inherited dementia FTDP-17. Nature. 1998;393(6686):702-705. doi:10.1038/31508 9641683

[4] DeJesus-Hernandez M, Mackenzie IR, Boeve BF, et al. Expanded GGGGCC hexanucleotide repeat in noncoding region of C9ORF72 causes chromosome 9p-linked FTD and ALS. Neuron. 2011;72(2):245-256. doi:10.1016/j.neuron.2011.09.011 21944778 PMC3202986

[5] Van Deerlin VM, Sleiman PM, Martinez-Lage M, et al. Common variants at 7p21 are associated with frontotemporal lobar degeneration with TDP-43 inclusions. Nat Genet. 2010;42(3):234-239. doi:10.1038/ng.536 20154673 PMC2828525

[6] Pottier C, Zhou X, Perkerson RB 3rd, et al. Potential genetic modifiers of disease risk and age at onset in patients with frontotemporal lobar degeneration and GRN mutations: a genome-wide association study. Lancet Neurol. 2018;17(6):548-558. doi:10.1016/S1474-4422(18)30126-1 29724592 PMC6237181

[7] van der Zee J, Van Langenhove T, Kleinberger G, et al. TMEM106B is associated with frontotemporal lobar degeneration in a clinically diagnosed patient cohort. Brain. 2011;134(Pt 3):808-815. doi:10.1093/brain/awr007 21354975 PMC3044834

[8] Finch N, Carrasquillo MM, Baker M, et al. TMEM106B regulates progranulin levels and the penetrance of FTLD in GRN mutation carriers. Neurology. 2011;76(5):467-474. doi:10.1212/WNL.0b013e31820a0e3b 21178100 PMC3034409

[9] Perneel J, Manoochehri M, Huey ED, Rademakers R, Goldman J. Case report: TMEM106B haplotype alters penetrance of GRN mutation in frontotemporal dementia family. Front Neurol. 2023;14:1160248. doi:10.3389/fneur.2023.1160248 37077569 PMC10106611

[10] van Blitterswijk M, Mullen B, Nicholson AM, et al. TMEM106B protects C9ORF72 expansion carriers against frontotemporal dementia. Acta Neuropathol. 2014;127(3):397-406. doi:10.1007/s00401-013-1240-4 24385136 PMC3944829

[11] Adams HH, Verhaaren BF, Vrooman HA, et al. TMEM106B influences volume of left-sided temporal lobe and interhemispheric structures in the general population. Biol Psychiatry. 2014;76(6):503-508. doi:10.1016/j.biopsych.2014.03.006 24731779

[12] Yu L, De Jager PL, Yang J, Trojanowski JQ, Bennett DA, Schneider JA. The TMEM106B locus and TDP-43 pathology in older persons without FTLD. Neurology. 2015;84(9):927-934. doi:10.1212/WNL.0000000000001313 25653292 PMC4351662

[13] Rhinn H, Abeliovich A. Differential aging analysis in human cerebral cortex identifies variants in TMEM106B and GRN that regulate aging phenotypes. Cell Syst. 2017;4(4):404-415 e5. doi:10.1016/j.cels.2017.02.009 28330615

[14] Li Z, Farias FHG, Dube U, et al. The TMEM106B FTLD-protective variant, rs1990621, is also associated with increased neuronal proportion. Acta Neuropathol. 2020;139(1):45-61. doi:10.1007/s00401-019-02066-0 31456032 PMC6942643

[15] Harding SR, Bocchetta M, Gordon E, et al. The TMEM106B risk allele is associated with lower cortical volumes in a clinically diagnosed frontotemporal dementia cohort. J Neurol Neurosurg Psychiatry. 2017;88(11):997-+. doi:998. 10.1136/jnnp-2017-315641 28446602 PMC5740537

[16] Premi E, Grassi M, van Swieten J, et al. Cognitive reserve and TMEM106B genotype modulate brain damage in presymptomatic frontotemporal dementia: a GENFI study. Brain. 2017;140(6):1784-1791. doi:10.1093/brain/awx103 28460069 PMC5445253

[17] Tropea TF, Mak J, Guo MH, et al. TMEM106B Effect on cognition in Parkinson disease and frontotemporal dementia. Ann Neurol. 2019;85(6):801-811. doi:10.1002/ana.25486 30973966 PMC6953172

[18] Rutherford NJ, Carrasquillo MM, Li M, et al. TMEM106B risk variant is implicated in the pathologic presentation of Alzheimer disease. Neurology. 2012;79(7):717-718. doi:10.1212/WNL.0b013e318264e3ac 22855871 PMC3467659

[19] Vass R, Ashbridge E, Geser F, et al. Risk genotypes at TMEM106B are associated with cognitive impairment in amyotrophic lateral sclerosis. Acta Neuropathol. 2011;121(3):373-380. doi:10.1007/s00401-010-0782-y 21104415 PMC3095217

[20] Manini A, Ratti A, Brusati A, et al. TMEM106B acts as a modifier of cognitive and motor functions in amyotrophic lateral sclerosis. Int J Mol Sci. 2022;23(16):9276. 10.3390/ijms23169276 36012536 PMC9408885

[21] Boeve B, Bove J, Brannelly P, et al. The longitudinal evaluation of familial frontotemporal dementia subjects protocol: framework and methodology. Alzheimers Dement. 2020;16(1):22-36. doi:10.1016/j.jalz.2019.06.4947 31636026 PMC6949411

[22] Ramos EM, Dokuru DR, Van Berlo V, et al. Genetic screening of a large series of North American sporadic and familial frontotemporal dementia cases. Alzheimers Dement. 2020;16(1):118-130. doi:10.1002/alz.12011 31914217 PMC7199807

[23] Ramos EM, Wojta K, Yang Z, et al. Inferring genetic relatedness in a large, multisite frontotemporal dementia series: data from the ALLFTD consortium. Alzheimer's Demen. 2023/06/01 2023;19(S1):e068040. doi:10.1002/alz.068040

[24] Staffaroni AM, Quintana M, Wendelberger B, et al. Temporal order of clinical and biomarker changes in familial frontotemporal dementia. Nat Med. 2022;28(10):2194-2206. doi:10.1038/s41591-022-01942-9 36138153 PMC9951811

[25] Sled JG, Zijdenbos AP, Evans AC. A nonparametric method for automatic correction of intensity nonuniformity in MRI data. IEEE Trans Med Imaging. 1998;17(1):87-97. doi:10.1109/42.668698 9617910

[26] Accessed April 4, 2024. fil.ion.ucl.ac.uk/spm

[27] Ashburner J, Friston KJ. Unified segmentation. Neuroimage. 2005;26(3):839-851. doi:10.1016/j.neuroimage.2005.02.018 15955494

[28] Ashburner J, Friston KJ. Diffeomorphic registration using geodesic shooting and Gauss-Newton optimisation. Neuroimage. 2011;55(3):954-967. doi:10.1016/j.neuroimage.2010.12.049 21216294 PMC3221052

[29] Desikan RS, Segonne F, Fischl B, et al. An automated labeling system for subdividing the human cerebral cortex on MRI scans into gyral based regions of interest. Neuroimage. 2006;31(3):968-980. doi:10.1016/j.neuroimage.2006.01.021 16530430

[30] Staffaroni AM, Asken BM, Casaletto KB, et al. Development and validation of the uniform data set (v3.0) executive function composite score (UDS3-EF). Alzheimers Dement. 2021;17(4):574-583. doi:10.1002/alz.12214 33215852 PMC8044003

[31] Asken BM, Ljubenkov PA, Staffaroni AM, et al. Plasma inflammation for predicting phenotypic conversion and clinical progression of autosomal dominant frontotemporal lobar degeneration. J Neurol Neurosurg Psychiatry. 2023;94(7):541-549. doi:10.1136/jnnp-2022-330866 36977552 PMC10313977

[32] Gendron TF, Heckman MG, White LJ, et al. Comprehensive cross-sectional and longitudinal analyses of plasma neurofilament light across FTD spectrum disorders. Cell Rep Med. 2022;3(4):100607. doi:10.1016/j.xcrm.2022.100607 35492244 PMC9044101

[33] Miyagawa T, Brushaber D, Syrjanen J, et al. Use of the CDR® plus NACC FTLD in mild FTLD: data from the ARTFL/LEFFTDS consortium. Alzheimers Dement. 2020;16(1):79-90. doi:10.1016/j.jalz.2019.05.013 31477517 PMC6949373

[34] Accessed April 4, 2024. allftd.org/data

[35] Bocchetta M, Gordon E, Cardoso MJ, et al. Thalamic atrophy in frontotemporal dementia - not just a C9orf72 problem. Neuroimage Clin. 2018;18:675-681. doi:10.1016/j.nicl.2018.02.019 29876259 PMC5988457

[36] Lee SE, Sias AC, Kosik EL, et al. Thalamo-cortical network hyperconnectivity in preclinical progranulin mutation carriers. Neuroimage Clin. 2019;22:101751. doi:10.1016/j.nicl.2019.101751 30921613 PMC6438992

[37] Lui H, Zhang J, Makinson SR, et al. Progranulin deficiency promotes circuit-specific synaptic pruning by microglia via complement activation. Cell. 2016;165(4):921-935. doi:10.1016/j.cell.2016.04.001 27114033 PMC4860138

[38] Marsan E, Velmeshev D, Ramsey A, et al. Astroglial toxicity promotes synaptic degeneration in the thalamocortical circuit in frontotemporal dementia with GRN mutations. J Clin Invest. 2023;133(6):e164919. doi:10.1172/JCI164919 36602862 PMC10014110

[39] Rohrer JD, Nicholas JM, Cash DM, et al. Presymptomatic cognitive and neuroanatomical changes in genetic frontotemporal dementia in the Genetic Frontotemporal dementia Initiative (GENFI) study: a cross-sectional analysis. Lancet Neurol. 2015;14(3):253-262. doi:10.1016/S1474-4422(14)70324-2 25662776 PMC6742501

[40] Rojas JC, Wang P, Staffaroni AM, et al. Plasma neurofilament light for prediction of disease progression in familial frontotemporal lobar degeneration. Neurology 2021;96(18):e2296–e2312. doi:10.1212/WNL.0000000000011848 33827960 PMC8166434

[41] Staffaroni AM, Goh SYM, Cobigo Y, et al. Rates of brain atrophy across disease stages in familial frontotemporal dementia Associated with MAPT, GRN, and C9orf72 pathogenic variants. JAMA Netw Open, 2020;3(10):e2022847. doi:10.1001/jamanetworkopen.2020.22847 33112398 PMC7593814

[42] White CC, Yang HS, Yu L, et al. Identification of genes associated with dissociation of cognitive performance and neuropathological burden: multistep analysis of genetic, epigenetic, and transcriptional data. PLoS Med. 2017;14(4):e1002287. doi:10.1371/journal.pmed.1002287 28441426 PMC5404753

[43] Rostgaard N, Roos P, Budtz-Jorgensen E, et al. TMEM106B and ApoE polymorphisms in CHMP2B-mediated frontotemporal dementia (FTD-3). Neurobiol Aging. 2017;59:221 e1-e221 e7. doi:10.1016/j.neurobiolaging.2017.06.026 28888721

[44] Aoki N, Murray ME, Ogaki K, et al. Hippocampal sclerosis in Lewy body disease is a TDP-43 proteinopathy similar to FTLD-TDP Type A. Acta Neuropathol. 2015;129(1):53-64. doi:10.1007/s00401-014-1358-z 25367383 PMC4282950

[45] Neumann M, Perneel J, Cheung S, et al. Limbic-predominant age-related TDP-43 proteinopathy (LATE-NC) is associated with abundant TMEM106B pathology. Acta Neuropathol. 2023;146(1):163-166. doi:10.1007/s00401-023-02580-2 37171635

[46] Cook C, Zhang YJ, Xu YF, Dickson DW, Petrucelli L. TDP-43 in neurodegenerative disorders. Expert Opin Biol Ther. 2008;8(7):969-978. doi:10.1517/14712598.8.7.969 18549326 PMC2855963

[47] Perneel J, Rademakers R. Identification of TMEM106B amyloid fibrils provides an updated view of TMEM106B biology in health and disease. Acta Neuropathol. 2022;144(5):807-819. doi:10.1007/s00401-022-02486-5 36056242 PMC9547799

[48] Lee JY, Harney DJ, Teo JD, et al. The major TMEM106B dementia risk allele affects TMEM106B protein levels, fibril formation, and myelin lipid homeostasis in the ageing human hippocampus. Mol Neurodegener. 2023;18(1):63. doi:10.1186/s13024-023-00650-3 37726834 PMC10510131

[49] Marks JD, Ayuso VE, Carlomagno Y, et al. TMEM106B core deposition associates with TDP-43 pathology and is increased in risk SNP carriers for frontotemporal dementia. Sci Transl Med. 2024;16(730):eadf9735. doi:10.1126/scitranslmed.adf9735 38232138 PMC10841341

[50] T Vicente C, Perneel J, Wynants S, et al. C-terminal TMEM106B fragments in human brain correlate with disease-associated TMEM106B haplotypes. Brain. 2023;146(10):4055-4064. doi:10.1093/brain/awad133 37100087 PMC10545506

[51] Busch JI, Unger TL, Jain N, Tyler Skrinak R, Charan RA, Chen-Plotkin AS. Increased expression of the frontotemporal dementia risk factor TMEM106B causes C9orf72-dependent alterations in lysosomes. Hum Mol Genet. 2016;25(13):2681-2697. doi:10.1093/hmg/ddw127 27126638 PMC5181637

[52] Staffaroni AM, Bajorek L, Casaletto KB, et al. Assessment of executive function declines in presymptomatic and mildly symptomatic familial frontotemporal dementia: NIH-EXAMINER as a potential clinical trial endpoint. Alzheimers Dement. 2020;16(1):11-21. doi:10.1016/j.jalz.2019.01.012 31914230 PMC6842665

